# Generalized fixed point results of rational type contractions in partially ordered metric spaces

**DOI:** 10.1186/s13104-021-05801-7

**Published:** 2021-10-15

**Authors:** N. Seshagiri Rao, K. Kalyani

**Affiliations:** 1 Department of Applied Mathematics, School of Applied Natural Sciences, Adama Science and Technology University, Post Box No. 1888, Adama, Ethiopia; 2 Department of Mathematics, Vignan’s Foundation for Science, Technology and Research, Vadlamudi, 522213 Andhra Pradesh India

**Keywords:** Partially ordered metric spaces, Generalized rational contractions, Fixed point, Ordered relation, Integral contractions, 54H25, 47H10

## Abstract

**Objectives:**

We investigated the existence and uniqueness of a fixed point for the mapping satisfying generalized rational type contraction conditions in metric space endowed with partial order. Suitable examples are presented to justify the results obtained.

**Result:**

Some new fixed point results have been obtained for a mapping fulfilling generalized contractions. The uniqueness of the fixed point is also the part of the study based on an ordered relation. One example is given for a result which is not valid in the usual metric space.

## Introduction

First the idea of fixed point theory was introduced by H.Poincare in 1886. Subsequently M.Frechet in 1906 has given the fixed point theorem in terms of taking distance between the points and also the corresponding images of the operator at those points in metric spaces. Later in 1922, Banach has proven a fixed theorem for a contraction mapping in complete metric space. This principle plays a crucial role in several branches of mathematics. It is an important tool for finding the solutions of many existing results in nonlinear analysis. Besides, this renowned classical theorem offers an iteration method through that we are able to acquire higher approximation to the fixed point. This result has rendered a key role in finding systems of linear algebraic equations involving iteration method. Iteration procedures are using in every branch of applied mathematics, convergence proof and also in estimating the process of errors, very often by an application of Banach’s fixed point theorem.

Since then several authors have generalized this classical Banach’s contraction theorem in an usual metric space and extensively reported in their work by taking various contraction conditions on the mappings, the readers may refer to [1–12]. Moreover, various generalizations of this result have been obtained by weakening its hypothesis in numerous spaces like rectangular metric spaces, pseudo metric spaces, fuzzy metric spaces, quasi metric spaces, quasi semi-metric areas, probabilistic metric spaces, *D*-metric spaces, *G*-metric spaces, *F*-metric spaces, cone metric spaces, some of which can be found in [13–28]. rkA lot of work on the results of fixed points, common fixed points, coupled fixed points in partly ordered metric spaces with different topological properties involved can be found from [29–41]. Some generalized fixed points results of monotone mappings in partially ordered *b*-metric spaces have been investigated by Seshagiri Rao et al. [43, 46, 47], Kalyani et al. [42, 45, 48] and Belay Mitiku et al. [44]. Acar [49] explored some fixed point results of *F*-contraction for multivalued integral type mapping on a complete metric space. Recently, the notation of Cirić type rational graphic $(Y, \Lambda )$-contraction pair mappings have been used and produced some new common fixed point results on partial *b*-metric spaces endowed with a directed graph *G* by Eskandar et al. [50].

The aim of this paper is to prove some fixed point results of a mapping in the frame work of a metric space endowed with partial order satisfying generalized contractive conditions of rational kind. The uniqueness of a fixed point is discussed through an ordered relation in a partially ordered metric space. Also, the conferred results generalize and extend a few well-known results of [20, 26] in the literature. Appropriate examples are highlighted to support the prevailing results.

## Preliminaries

We start this section with the following subsequent definitions which are used frequently in our study.

### Definition 1

[36] The triple $({\mathscr {Q}},\varrho ,\preceq )$ is called partially ordered metric spaces if $({\mathscr {Q}},\preceq )$ could be a partial ordered set and $({\mathscr {Q}},\varrho )$ be a metric space.

### Definition 2

[36] If $\varrho$ is complete metric, then $({\mathscr {Q}},\varrho ,\preceq )$ is called complete partially ordered metric space.

### Definition 3

[36] A partially ordered metric space $({\mathscr {Q}},\varrho ,\preceq )$ is called an ordered complete (OC), if for every convergent sequence $\{\mho _n\} \subset {\mathscr {Q}}$, the subsequent condition holds: either

- if a non-increasing sequence $\mho _n \rightarrow \mho \in {\mathscr {Q}}$, then $\mho \preceq \mho _n$, for all $n \in {\mathbb {N}}$, that is, $\mho = \inf \{\mho _n\}$, or
- if $\mho _n \in {\mathscr {Q}}$ is a non-decreasing sequence such that $\mho _n\rightarrow \mho$ implies that $\mho _n \preceq \mho$, for all $n \in {\mathbb {N}}$, that is, $\mho = \sup \{\mho _n\}$.

### Definition 4

[36] A map ${\mathscr {I}}:{\mathscr {Q}} \rightarrow {\mathscr {Q}}$ is a non-decreasing, if for every $\mho ,\varepsilon \in {\mathscr {Q}}$ with $\mho <\varepsilon$ implies that ${\mathscr {I}}\mho \ge {\mathscr {I}}\varepsilon$.

## Main text

We begin this section with the subsequent result.

### Theorem 1

*Let* $({\mathscr {Q}},\varrho ,\preceq )$* be a complete partially ordered metric space. Suppose a self-map* ${\mathscr {I}}$* on* ${\mathscr {Q}}$* is continuous, non-decreasing and satisfies the contraction condition*

1 $$\begin{aligned} \begin{aligned} \varrho ({\mathscr {I}}\mho ,{\mathscr {I}}\varepsilon )&\le ~\alpha \frac{\varrho (\mho ,{\mathscr {I}}\mho )\varrho (\varepsilon ,{\mathscr {I}}\varepsilon )}{\varrho (\mho ,\varepsilon )} + \beta [\varrho (\mho ,{\mathscr {I}}\mho )+\varrho (\varepsilon ,{\mathscr {I}}\varepsilon )] \\&\quad + \,\gamma \varrho (\mho ,\varepsilon )+ {\mathscr {L}}~ \min \{\varrho (\mho ,{\mathscr {I}}\varepsilon ),\varrho (\varepsilon ,{\mathscr {I}}\mho )\}, \end{aligned} \end{aligned}$$

*for any* $\mho \ne \varepsilon \in {\mathscr {Q}}$ *with* $\mho \preceq \varepsilon$,* where* ${\mathscr {L}} \ge 0$,* and* $\alpha , \beta , \gamma \in [0,1)$ *with* $0\le \alpha +2\beta +\gamma <1$. *If* $\mho _0\preceq {\mathscr {I}}\mho _0$* for certain* $\mho _0 \in {\mathscr {Q}}$,* then* ${\mathscr {I}}$* has a fixed point*.

### Proof

Define a sequence, $\mho _{n+1}={\mathscr {I}}\mho _n$ for $\mho _0 \in {\mathscr {Q}}$. If $\mho _{n_0}=\mho _{n_0+1}$ for certain $n_0 \in {\mathbb {N}}$, then $\mho _{n_0}$ is a fixed point of ${\mathscr {I}}$. Assume that $\mho _n \ne \mho _{n+1}$ for each *n*. But $\mho _0 \preceq {\mathscr {I}}\mho _0$ and ${\mathscr {I}}$ is non-decreasing as by induction we obtain that

2 $$\begin{aligned} \mho _0\preceq \mho _1\preceq \mho _2 \preceq ...\preceq \mho _n\preceq \mho _{n+1} \preceq ...~. \end{aligned}$$

Now

$$\begin{aligned} \begin{aligned} \varrho (\mho _{n+1},\mho _n)&= \varrho ({\mathscr {I}}\mho _n,{\mathscr {I}}\mho _{n-1})\\&\le \alpha \frac{\varrho (\mho _n,{\mathscr {I}}\mho _n)~\varrho (\mho _{n-1},{\mathscr {I}}\mho _{n-1})}{\varrho (\mho _n,\mho _{n-1})} + \beta [\varrho (\mho _n,{\mathscr {I}}\mho _n)+\varrho (\mho _{n-1},{\mathscr {I}}\mho _{n-1})] \\&\quad +\,\gamma \varrho (\mho _n,\mho _{n-1}) + {\mathscr {L}}~ \min \{\varrho (\mho _n,{\mathscr {I}}\mho _{n-1}),\varrho (\mho _{n-1},{\mathscr {I}}\mho _n)\}, \end{aligned} \end{aligned}$$

which infer that

$$\begin{aligned} \varrho (\mho _{n+1},\mho _n) \le \left( \frac{\beta +\gamma }{1-\alpha -\beta }\right) \varrho (\mho _n,\mho _{n-1})\le ....\le \left( \frac{\beta +\gamma }{1-\alpha -\beta }\right) ^n\varrho (\mho _1,\mho _0). \end{aligned}$$

Furthermore, the triangular inequality of *d*, we have for $m\ge n$,

3 $$\begin{aligned} \begin{aligned} \varrho (\mho _n,\mho _m)&= \varrho (\mho _n,\mho _{n+1})+\varrho (\mho _{n+1},\mho _{n+2})+....+\varrho (\mho _{m-1},\mho _m)\\&\le ~\left( \varkappa ^n+\varkappa ^{n+1}+...+\varkappa ^{m-1} \right) \varrho (\mho _0, T\mho _0)\\&\le ~\frac{\varkappa ^n}{1-\varkappa }\varrho (\mho _1, \mho _0), \end{aligned} \end{aligned}$$

where $\varkappa =\frac{\beta +\gamma }{1-\alpha -\beta }$. As $n \rightarrow \infty$ in Eq. (3), we obtain $\varrho (\mho _n,\mho _m)=0$. This shows that $\{\mho _n\} \in {\mathscr {Q}}$ is a Cauchy sequence and then $\mho _n \rightarrow \zeta \in {\mathscr {Q}}$ by its completeness. Besides, the continuity of ${\mathscr {I}}$ implies that

$$\begin{aligned} {\mathscr {I}}\zeta = {\mathscr {I}}\left( \lim \limits _{n \rightarrow \infty }\mho _n \right) =\lim \limits _{n \rightarrow \infty }T\mho _n =\lim \limits _{n \rightarrow \infty }\mho _{n+1} =\zeta . \end{aligned}$$

Therefore, $\zeta$ is a fixed point of ${\mathscr {I}}$ in ${\mathscr {Q}}$. $\square$

Extracting the continuity of a map ${\mathscr {I}}$ in Theorem 1, we have the below result.

### Theorem 2

*Suppose* $({\mathscr {Q}},\varrho ,\preceq )$* is a complete partially ordered metric space. A non-decreasing mapping* ${\mathscr {I}}$* has a fixed point, if it satisfies the following assumption with* $\mho _0\preceq {\mathscr {I}}\mho _0$* for certain* $\mho _0 \in {\mathscr {Q}}$.

4 $$\begin{aligned} \text {If a nondecreasing sequence}~ \{\mho _n\} \rightarrow \mho ~ \text {in}~ {\mathscr {Q}},~ \text {then}~ \mho =\sup \{\mho _n\}. \end{aligned}$$

### Proof

The required proof can be obtained by following the proof of Theorem 8. $\square$

### Example 1

Let ${\mathscr {Q}}_1=\{(2,0), (0,2)\} \subseteq {\mathbb {R}}^2$ with the Euclidean distance $\varrho$. Define the partial order in ${\mathscr {Q}}_1$ as below

$$\begin{aligned} (\mho _1,\zeta _1)\le (\mho _2,\zeta _2)~\text {if and only if}~ \mho _1\le \mho _2 ~\text {and}~\zeta _1 \le \zeta _2. \end{aligned}$$

It is evident that, $({\mathscr {Q}}_1, \varrho , \le )$ is a complete partially ordered metric space and a map ${\mathscr {I}}(\mho ,\zeta )=(\mho ,\zeta )$ is non-decreasing and continuous. Consider

$$\begin{aligned} \begin{aligned} \varrho ({\mathscr {I}}(\mho _1,\zeta _1),{\mathscr {I}}(\mho _2,\zeta _2))&\le \gamma \varrho ((\mho _1,\zeta _1),(\mho _2,\zeta _2))\\&\le \alpha \frac{\varrho ((\mho _1,\zeta _1),{\mathscr {I}}(\mho _1,\zeta _1))\varrho ((\mho _2,\zeta _2),{\mathscr {I}}(\mho _2,\zeta _2))}{\varrho ((\mho _1,\zeta _1),(\mho _2,\zeta _2))}\\ {}&+\,\beta \left[ \varrho ((\mho _1,\zeta _1),{\mathscr {I}}(\mho _1,\zeta _1))+\varrho ((\mho _2,\zeta _2),{\mathscr {I}}(\mho _2,\zeta _2))\right] +\gamma \varrho ((\mho _1,\zeta _1),(\mho _2,\zeta _2))\\&\quad +\,{\mathscr {L}} \min \{\varrho ((\mho _1,\zeta _1),{\mathscr {I}}(\mho _2,\zeta _2)),\varrho ((\mho _2,\zeta _2),{\mathscr {I}}(\mho _1,\zeta _1))\}, \end{aligned} \end{aligned}$$

which holds for every $\alpha , \beta , \gamma \in [0,1)$ with $0 \le \alpha +2\beta +\gamma <1$ and any ${\mathscr {L}} \ge 0$. Also note that the elements of ${\mathscr {Q}}_1$ are comparable to themselves only. Furthermore, $(0,2)\le {\mathscr {I}}((0,2))$. Therefore, all assumptions of Theorem 1 are met and ${\mathscr {I}}$ has two fixed points (2, 0), (0, 2).

### Example 2

The identity mapping ${\mathscr {I}}$ has an infinite number of fixed points in ${\mathscr {Q}}_2=\{(\mho ,-\mho ), \mho \in {\mathbb {R}}\}$, as any two distinct elements are not comparable in ${\mathscr {Q}}_2$ with usual order and the Euclidean distance ($\varrho$).

### Theorem 3

*The unique fixed point of* ${\mathscr {I}}$* in Theorems* 1* and* 2* can be found from the condition* (11)* stated below*.

### Example 3

Define a self map ${\mathscr {I}}: {\mathscr {Q}} \rightarrow {\mathscr {Q}}$, where ${\mathscr {Q}}=[0,1]$ with usual metric and usual order $\varepsilon \le \mho$, for $\mho , \varepsilon \in {\mathscr {Q}}$ by

$$\begin{aligned} \begin{aligned} \varrho (\mho ,\varepsilon )= {\left\{ \begin{array}{ll} \frac{\mho }{24}, \quad & if~ \mho \in \Big [0,\frac{1}{4}\Big ] \\ \frac{\mho }{12}-\frac{1}{96}, \quad & if \mho \in \Big (\frac{1}{4}, 1\Big ]. \end{array}\right. } \end{aligned} \end{aligned}$$

Then ${\mathscr {I}}$ has a unique fixed point in ${\mathscr {Q}}$.

### Proof

We will discuss the proof thoroughly by the subsequent cases.

**Case: 1** If $\mho ,\varepsilon \in [0,\frac{1}{4})$, then

$$\begin{aligned} \begin{aligned} \varrho ({\mathscr {I}}\mho ,{\mathscr {I}}\varepsilon )&= \frac{1}{24}|\mho -\varepsilon |\le \frac{1}{10}|\mho -\varepsilon |=\frac{1}{10} ~\varrho (\mho ,\varepsilon )\\&\le \alpha \frac{\varrho (\mho ,{\mathscr {I}}\mho )\varrho (\varepsilon ,{\mathscr {I}}\varepsilon )}{\varrho (\mho ,\varepsilon )} + \beta [\varrho (\mho ,{\mathscr {I}}\mho )+\varrho (\varepsilon ,{\mathscr {I}}\varepsilon )]\\&\quad + \frac{1}{10} \varrho (\mho ,\varepsilon )+ {\mathscr {L}} \min \{\varrho (\mho ,{\mathscr {I}}\varepsilon ),\varrho (\varepsilon ,{\mathscr {I}}\mho )\}, \end{aligned} \end{aligned}$$

this inequality is true for every $\alpha , \beta \in [0,1)$ with $0\le \alpha +2\beta +\gamma <1$, and ${\mathscr {L}} \ge 0$. Consequently all conditions of Theorem 1 are fulfilled in this case.

**Case: 2** If $\mho ,\varepsilon \in (\frac{1}{4}, 1]$, then

$$\begin{aligned} \begin{aligned} \varrho ({\mathscr {I}}\mho ,{\mathscr {I}}\varepsilon )&= \frac{1}{12}|\mho -\varepsilon |\le \frac{1}{10}|\mho -\varepsilon |=\frac{1}{10}~\varrho (\mho ,\varepsilon )\\&\le \alpha \frac{\varrho (\mho ,{\mathscr {I}}\mho )\varrho (\varepsilon ,{\mathscr {I}}\varepsilon )}{\varrho (\mho ,\varepsilon )} + \beta [\varrho (\mho ,{\mathscr {I}}\mho )+\varrho (\varepsilon ,{\mathscr {I}}\varepsilon )] \\&\quad + \frac{1}{10} \varrho (\mho ,\varepsilon )+ {\mathscr {L}}\min \{\varrho (\mho ,{\mathscr {I}}\varepsilon ),\varrho (\varepsilon ,{\mathscr {I}}\mho )\}, \end{aligned} \end{aligned}$$

this inequality holds for any ${\mathscr {L}} \ge 0$ and every $\alpha , \beta \in [0,1)$ with $0\le \alpha +2\beta +\gamma <1$. Thus, all assumptions in Theorem 1 are met.

**Case: 3** If $\varepsilon \in [0,\frac{1}{4})$ and $\mho \in (\frac{1}{4}, 1]$, then we have $\frac{1}{96}|4\mho -1|\le \frac{1}{96}$, $\frac{23}{96}\le \varrho (\mho ,{\mathscr {I}}\varepsilon )=|\mho -\frac{\varepsilon }{24}|\le 1$, and $\frac{1}{96}\le \varrho (\varepsilon ,{\mathscr {I}}\mho )=|\frac{\mho }{12}-\frac{1}{96}-\varepsilon |\le \frac{23}{96}$. Therefore,

$$\begin{aligned} \begin{aligned} \varrho ({\mathscr {I}}\mho ,{\mathscr {I}}\varepsilon )&= \left| \frac{\mho }{12}-\frac{1}{96}-\frac{\varepsilon }{24}\right| \\&\le \frac{1}{24}|\mho -\varepsilon |+\frac{1}{96}|4\mho -1|\\&\le \alpha \frac{\varrho (\mho ,{\mathscr {I}}\mho )\varrho (\varepsilon ,{\mathscr {I}}\varepsilon )}{\varrho (\mho ,\varepsilon )} + \beta [\varrho (\mho ,{\mathscr {I}}\mho )+\varrho (\varepsilon ,{\mathscr {I}}\varepsilon )]\\&\quad + \frac{1}{10} \varrho (\mho ,\varepsilon )+ {\mathscr {L}} \min \{\varrho (\mho ,{\mathscr {I}}\varepsilon ),\varrho (\varepsilon ,{\mathscr {I}}\mho )\}, \end{aligned} \end{aligned}$$

holds for any ${\mathscr {L}} \ge 0$ and for any $\alpha , \beta \in [0,1)$ with $0\le \alpha +2\beta +\gamma <1$. Since all other hypotheses of Theorem 1 are satisfied, as a result $0 \in {\mathscr {Q}}$ is a unique fixed point of ${\mathscr {I}}$. $\square$

### Corollary 1

*Suppose* $({\mathscr {Q}},\varrho ,\preceq )$* is a complete partially ordered metric space. A non-decreasing continuous self-map* ${\mathscr {I}}$* on* ${\mathscr {Q}}$* satisfies*

5 $$\begin{aligned}&\varrho ({\mathscr {I}}\mho ,{\mathscr {I}}\varepsilon )\le \alpha \frac{\varrho (\mho ,{\mathscr {I}}\mho )\varrho (\varepsilon ,{\mathscr {I}}\varepsilon )}{\varrho (\mho ,\varepsilon )}\nonumber \\&\quad +\, \beta [\varrho (\mho ,{\mathscr {I}}\mho )+\varrho (\varepsilon ,{\mathscr {I}}\varepsilon )] + \gamma \varrho (\mho ,\varepsilon ), \end{aligned}$$

*for any* $\mho \ne \varepsilon \in {\mathscr {Q}}$* with* $\mho \preceq \varepsilon$,* and some* $\alpha , \beta , \gamma \in [0,1)$* with* $0\le \alpha +2\beta +\gamma <1$.* If* $\mho _0\preceq {\mathscr {I}}\mho _0$,* for* $\mho _0 \in {\mathscr {Q}}$,* then* ${\mathscr {I}}$* has a fixed point*.

### Proof

Put ${\mathscr {L}}=0$ in Theorem 1. $\square$

### Example 4

Define a metric $\varrho$ on ${\mathscr {Q}}=[0,\infty )$ by

$$\begin{aligned} \begin{aligned} \varrho (\mho ,\varepsilon )= {\left\{ \begin{array}{ll} max \{\mho ,\varepsilon \},\quad &{} if~ \mho \ne \varepsilon \\ 0,\quad &{} if~ \mho = \varepsilon . \end{array}\right. } \end{aligned} \end{aligned}$$

Also, let us define ${\mathscr {I}}:{\mathscr {Q}} \rightarrow {\mathscr {Q}}$ by

$$\begin{aligned} \begin{aligned} {\mathscr {I}}\mho = {\left\{ \begin{array}{ll} \frac{\mho }{10(1+\mho )}, \quad &{} if~ 0 \le \mho \le 5,\\ \frac{\mho }{20}, \quad &{} if~ 5<\mho , \end{array}\right. } \end{aligned} \end{aligned}$$

with $\mho \preceq \varepsilon$ iff $\mho \le \varepsilon$. Then from Corollary 1, ${\mathscr {I}}$ has a fixed point.

### Proof

Consider the subsequent attainable cases to debate the proof of the theory.

**Case: 1** If $0\le \mho <\varepsilon \le 5$, then

$$\begin{aligned} \varrho ({\mathscr {I}}\mho ,{\mathscr {I}}\varepsilon )&=\max \{{\mathscr {I}}\mho ,{\mathscr {I}}\varepsilon \}=\max \Bigg \{\frac{\mho }{10(1+\mho )}, \frac{\varepsilon }{10(1+\varepsilon )}\Bigg \}\le \frac{2}{5}\varepsilon \\&=\frac{1}{5} \left( \mho +\varepsilon \right) +\frac{1}{5} \left( \mho +\varepsilon \right) =\frac{1}{5}\left( \frac{\mho \varepsilon }{\varepsilon }+\varepsilon \right) +\frac{1}{5} \left( \mho +\varepsilon \right) \\&=\frac{1}{5} ~~\Bigg [ \frac{\max \Big \{\mho ,\frac{\mho }{10(1+\mho )}\Big \}, \max \Big \{\varepsilon ,\frac{\varepsilon }{10(1+\varepsilon )}\Big \}}{\max \{\mho ,\varepsilon \}} \\&\quad + \left( \max \Bigg \{\mho ,\frac{\mho }{10(1+\mho )}\Bigg \}+\max \Bigg \{\varepsilon ,\frac{\varepsilon }{10(1+\varepsilon )}\Bigg \}\right) +\max \{\mho ,\varepsilon \}\Bigg ]\\&=\frac{1}{5} \left( \frac{\varrho (\mho ,{\mathscr {I}}\mho )\varrho (\varepsilon ,{\mathscr {I}}\varepsilon )}{\varrho (\mho ,\varepsilon )} + [\varrho (\mho ,{\mathscr {I}}\mho )+\varrho (\varepsilon ,{\mathscr {I}}\varepsilon )] + \varrho (\mho ,\varepsilon ) \right) , \end{aligned}$$

implies that,

$$\begin{aligned}&\varrho ({\mathscr {I}}\mho ,{\mathscr {I}}\varepsilon )\le \frac{1}{5} \frac{\varrho (\mho ,{\mathscr {I}}\mho )\varrho (\varepsilon ,{\mathscr {I}}\varepsilon )}{\varrho (\mho ,\varepsilon )}\\&\quad + \frac{1}{5} [\varrho (\mho ,{\mathscr {I}}\mho )+\varrho (\varepsilon ,{\mathscr {I}}\varepsilon )] +\frac{1}{5} \varrho (\mho ,\varepsilon ). \end{aligned}$$

**Case: 2** If $5< \mho <\varepsilon$, then

$$\begin{aligned} \varrho ({\mathscr {I}}\mho ,{\mathscr {I}}\varepsilon )&=\max \{{\mathscr {I}}\mho ,{\mathscr {I}}\varepsilon \}=\max \Bigg \{\frac{\mho }{20}, \frac{\varepsilon }{20}\Bigg \}=\frac{\varepsilon }{20}\le \frac{2}{5}\varepsilon \\&=\frac{1}{5} \left( \mho +\varepsilon \right) +\frac{1}{5} \left( \mho +\varepsilon \right) =\frac{1}{5}\left( \frac{\mho \varepsilon }{\varepsilon }+\varepsilon \right) +\frac{1}{5} \left( \mho +\varepsilon \right) \\&=\frac{1}{5} \left[ \frac{\max \Big \{\mho ,\frac{\mho }{20}\Big \}, \max \Big \{\varepsilon ,\frac{\varepsilon }{20}\Big \}}{\max \{\mho ,\varepsilon \}}+ \left[ \max \Big \{\mho ,\frac{\mho }{20}\Big \}+\max \Big \{\varepsilon ,\frac{\varepsilon }{20}\Big \}\right] +\max \{\mho ,\varepsilon \}\right] \\&=\frac{1}{5} \left( \frac{\varrho (\mho ,{\mathscr {I}}\mho )\varrho (\varepsilon ,{\mathscr {I}}\varepsilon )}{\varrho (\mho ,\varepsilon )} + [\varrho (\mho ,{\mathscr {I}}\mho )+\varrho (\varepsilon ,{\mathscr {I}}\varepsilon )] + \varrho (\mho ,\varepsilon ) \right) , \end{aligned}$$

which implies that,

$$\begin{aligned}&\varrho ({\mathscr {I}}\mho ,{\mathscr {I}}\varepsilon )\le \frac{1}{5} \frac{\varrho (\mho ,{\mathscr {I}}\mho )\varrho (\varepsilon ,{\mathscr {I}}\varepsilon )}{\varrho (\mho ,\varepsilon )}\\&\quad + \frac{1}{5} [\varrho (\mho ,{\mathscr {I}}\mho )+\varrho (\varepsilon ,{\mathscr {I}}\varepsilon )] +\frac{1}{5} \varrho (\mho ,\varepsilon ). \end{aligned}$$

**Case: 3** If $0\le \mho \le 5$ and $5<\varepsilon$, then

$$\begin{aligned} \varrho ({\mathscr {I}}\mho ,{\mathscr {I}}\varepsilon )&=\max \{{\mathscr {I}}\mho ,{\mathscr {I}}\varepsilon \}=\max \Big \{\frac{\mho }{10(1+\mho )}, \frac{\varepsilon }{20}\Big \}=\frac{\varepsilon }{20}\le \frac{2}{5}\varepsilon \\&=\frac{1}{5} \left( \mho +\varepsilon \right) +\frac{1}{5} \left( \mho +\varepsilon \right) =\frac{1}{5}\left( \frac{\mho \varepsilon }{\varepsilon }+\varepsilon \right) +\frac{1}{5} \left( \mho +\varepsilon \right) \\&=\frac{1}{5} \left[ \frac{\max \Big \{\mho ,\frac{\mho }{10(1+\mho )}\Big \}, \max \Big \{\varepsilon ,\frac{\varepsilon }{20}\Big \}}{\max \{\mho ,\varepsilon \}}+ \left[ \max \Big \{\mho ,\frac{\mho }{10(1+\mho )}\Big \}+\max \Big \{\varepsilon ,\frac{\varepsilon }{20}\Big \}\right] +\max \{\mho ,\varepsilon \}\right] \\&=\frac{1}{5} \left( \frac{\varrho (\mho ,{\mathscr {I}}\mho )\varrho (\varepsilon ,{\mathscr {I}}\varepsilon )}{\varrho (\mho ,\varepsilon )} + [\varrho (\mho ,{\mathscr {I}}\mho )+\varrho (\varepsilon ,{\mathscr {I}}\varepsilon )] + \varrho (\mho ,\varepsilon ) \right) . \end{aligned}$$

Therefore,

$$\begin{aligned}&\varrho ({\mathscr {I}}\mho ,{\mathscr {I}}\varepsilon )\le \frac{1}{5} \frac{\varrho (\mho ,{\mathscr {I}}\mho )\varrho (\varepsilon ,{\mathscr {I}}\varepsilon )}{\varrho (\mho ,\varepsilon )}\\&\quad + \frac{1}{5} [\varrho (\mho ,{\mathscr {I}}\mho )+\varrho (\varepsilon ,{\mathscr {I}}\varepsilon )] +\frac{1}{5} \varrho (\mho ,\varepsilon ). \end{aligned}$$

Subsequently, all conditions of Corollary 1 are fulfilled and hence the self mapping ${\mathscr {I}}$ has a fixed point $0 \in {\mathscr {Q}}$. $\square$

Apart from, if ${\mathscr {Q}}$ satisfies the conditions (4) and (11), then a mapping ${\mathscr {I}}$ has a fixed point and also it’s uniqueness in Corollary 1.

### Theorem 4

*Suppose* $({\mathscr {Q}},\varrho ,\preceq )$* is a complete partially ordered metric space. A non-decreasing self mapping* ${\mathscr {I}}$* is such that either* ${\mathscr {I}}$* is continuous or* ${\mathscr {Q}}$* satisfies the following condition in Theorems* 1* and* 2* and Corollary* 1,* then* ${\mathscr {I}}$* has a fixed point in* ${\mathscr {Q}}$,* for* $\mho _0 \in {\mathscr {Q}}$* such that* $\mho _0\succeq {\mathscr {I}}\mho _0$.

$$\begin{aligned} \text {If a nonincreasing sequence}~ \{\mho _n\} \rightarrow \mho ~ \text {in}~ {\mathscr {Q}},~ \text {then}~ \mho =\inf \{\mho _n\}. \end{aligned}$$

### Proof

The scheme of the proof is similar to the procedure of the proofs of the previous theorems. $\square$

In particular, there is an example where Theorem 1 (or Corollary 1) can be applied and not be valid in a complete metric space.

### Example 5

Let ${\mathscr {Q}}=\{(0,1),(1,0),(1,1)\}$ and, let the partial order relation on ${\mathscr {Q}}$ be $R=\{(\mho ,\mho ):\mho \in {\mathscr {Q}}\}$. Observe that the elements only in ${\mathscr {Q}}$ are comparable to themselves. Apart from, $({\mathscr {Q}},\varrho )$ is a complete metric space with the Euclidean distance ($\varrho$) while with regards $\le$ is a partially ordered set.

Define a map ${\mathscr {I}}:{\mathscr {Q}} \rightarrow {\mathscr {Q}}$ by

$$\begin{aligned} {\mathscr {I}}(0,1)=(1,0),~~{\mathscr {I}}(1,0)=(0,1),~~ {\mathscr {I}}(1,1)=(1,1), \end{aligned}$$

is a nondecreasing, continuous and, $(1,1)\le {\mathscr {I}}(1,1)=(1,1)$ for $(1,1) \in {\mathscr {Q}}$ and satisfy condition (1) (or(5)). As a result (1, 1) is a fixed point of ${\mathscr {I}}$.

Besides, for $\mho =(0,1)$, $\zeta =(1,0)$ in ${\mathscr {Q}}$, we have

$$\begin{aligned} \varrho ({\mathscr {I}}\mho ,{\mathscr {I}}\zeta )=\sqrt{2},~\varrho (\mho ,{\mathscr {I}}\zeta )=0,~\varrho (\zeta ,{\mathscr {I}}\mho )=0,~\varrho (\mho ,{\mathscr {I}}\mho )=\sqrt{2},~\varrho (\zeta ,{\mathscr {I}}\zeta )=\sqrt{2}, \end{aligned}$$

then

$$\begin{aligned} \begin{aligned} \varrho ({\mathscr {I}}\mho ,{\mathscr {I}}\zeta )=\sqrt{2}&\le \alpha \frac{\varrho (\mho ,{\mathscr {I}}\mho )~\varrho (\zeta ,{\mathscr {I}}\zeta )}{\varrho (\mho ,\zeta )} + \beta [\varrho (\mho ,{\mathscr {I}}\mho )+\varrho (\zeta ,{\mathscr {I}}\zeta )] + \gamma \varrho (\mho ,\zeta )\\&\le \alpha .\frac{\sqrt{2}.\sqrt{2}}{\sqrt{2}}+ \beta [\sqrt{2}+\sqrt{2}]. +\gamma .\sqrt{2}\\&=(\alpha +2\beta +\gamma ).\sqrt{2}, \end{aligned} \end{aligned}$$

which implies that, $\alpha +2\beta +\gamma \ge 1$. Accordingly, this example is not valid in the case of usual complete metrical space.

Also, notice here that ${\mathscr {I}}$ has a unique fixed point even though ${\mathscr {Q}}$ doesn’t satisfies the condition (11) stated below. Hence, as a result condition (11) is not necessary for the existence of the uniqueness of a fixed point.

In the next theorem, we set up the existence of a unique fixed point of a mapping ${\mathscr {I}}$ through assuming most effective the continuity of some iteration of it.

### Theorem 5

*If* ${\mathscr {I}}^p$* is continuous for some positive integer* *p in Theorem* 1,* then* ${\mathscr {I}}$* has a fixed point*.

### Proof

From Theorem 1, there is a Cauchy sequence $\{\mho _n\} \subset {\mathscr {Q}}$ such that $\{\mho _n\} \rightarrow \zeta \in {\mathscr {Q}}$ as a result its subsequence $\mho _{n_k} (n_k=kp)$ converges to the same point. Moreover,

$$\begin{aligned} {\mathscr {I}}^p\zeta = {\mathscr {I}}^p\left( \lim \limits _{n \rightarrow \infty }\mho _{n_k} \right) =\lim \limits _{n \rightarrow \infty }\mho _{n_{k+1}} =\zeta , \end{aligned}$$

which shows that $\zeta$ is a fixed point of ${\mathscr {I}}^p$. Next to claim that ${\mathscr {I}}\zeta =\zeta$. Assume *m* is the smallest among all positive integer so that ${\mathscr {I}}^m\zeta =\zeta$ and ${\mathscr {I}}^q\zeta \ne \zeta ~(q=1,2,3,..., m-1)$. If $m>1$, then

$$\begin{aligned} \begin{aligned} \varrho ({\mathscr {I}}\zeta ,\zeta )&=\varrho ({\mathscr {I}}\zeta ,{\mathscr {I}}^m\zeta )\\&\le \alpha \frac{\varrho (\zeta ,{\mathscr {I}}\zeta )\varrho ({\mathscr {I}}^{m-1}\zeta ,{\mathscr {I}}^m\zeta )}{\varrho (\zeta , {\mathscr {I}}^{m-1}\zeta )} + \beta [\varrho (\zeta ,{\mathscr {I}}\zeta )+\varrho ({\mathscr {I}}^{m-1} \zeta ,{\mathscr {I}}^m \zeta )]+ \gamma \varrho (\zeta ,{\mathscr {I}}^{m-1} \zeta ) \\&\quad +\, {\mathscr {L}} \min \{\varrho (\zeta , {\mathscr {I}}^m \zeta ),\varrho ({\mathscr {I}}^{m-1} \zeta ,{\mathscr {I}}\zeta )\}. \end{aligned} \end{aligned}$$

Therefore,

$$\begin{aligned} \varrho (\zeta , {\mathscr {I}}\zeta ) \le \left( \frac{\beta +\gamma }{1-\alpha -\beta }\right) \varrho (\zeta , T^{m-1}\zeta ). \end{aligned}$$

Regarding (1), we have

$$\begin{aligned} \begin{aligned} \varrho (\zeta ,{\mathscr {I}}^{m-1}\zeta )&=\varrho ({\mathscr {I}}^m\zeta ,{\mathscr {I}}^{m-1}\zeta )\\&\le \alpha \frac{\varrho ({\mathscr {I}}^{m-1}\zeta ,{\mathscr {I}}^m\zeta ).\varrho ({\mathscr {I}}^{m-2}\zeta ,{\mathscr {I}}^{m-1}\zeta )}{\varrho ({\mathscr {I}}^{m-1}\zeta ,{\mathscr {I}}^{m-2}\zeta )}\\&\quad + \,\beta [\varrho ({\mathscr {I}}^{m-1}\zeta ,{\mathscr {I}}^m\zeta )+\varrho ({\mathscr {I}}^{m-2} \zeta ,{\mathscr {I}}^{m-1} \zeta )] + \gamma \varrho ({\mathscr {I}}^{m-1}\zeta ,{\mathscr {I}}^{m-2} \zeta ) \\&\quad +\, {\mathscr {L}}~ \min \{\varrho ({\mathscr {I}}^{m-1}\zeta , {\mathscr {I}}^{m-1} \zeta ), \varrho ({\mathscr {I}}^{m-2} \zeta ,{\mathscr {I}}^m \zeta )\}. \end{aligned} \end{aligned}$$

By induction, we get

$$\begin{aligned}&\varrho (\zeta ,{\mathscr {I}}^{m-1}\zeta )= \varrho ({\mathscr {I}}^m\zeta ,{\mathscr {I}}^{m-1}\zeta ) \\&\quad \le \varkappa \varrho ({\mathscr {I}}^{m-1}\zeta ,{\mathscr {I}}^{m-2}\zeta ) \le ...\le \varkappa ^{m-1} \varrho ({\mathscr {I}}\zeta ,\zeta ), \end{aligned}$$

where $$\varkappa =\frac{\beta +\gamma }{1-\alpha -\beta }<1$$. Therefore,

$$\begin{aligned} \varrho ({\mathscr {I}}\zeta ,\zeta ) \le \varkappa ^{m} \varrho ({\mathscr {I}}\zeta ,\zeta )< \varrho ({\mathscr {I}}\zeta ,\zeta ), \end{aligned}$$

a contradiction. Hence, $${\mathscr {I}}\zeta =\zeta $$. $$\square $$

### Corollary 2

*If* ${\mathscr {I}}^p$* is continuous for some positive integer p*,* then* ${\mathscr {I}}$* has a fixed point in Corollary* 1.

### Proof

Put ${\mathscr {L}}=0$ in Theorem 5. $\square$

### Theorem 6

*Suppose* $({\mathscr {Q}},\varrho ,\preceq )$* is a complete partially ordered metric space and* ${\mathscr {I}}$* be a non-decreasing self map on* ${\mathscr {Q}}$.* Assume for some positive integer* *m*, ${\mathscr {I}}$* satisfies*

6 $$\begin{aligned} \begin{aligned} \varrho ({\mathscr {I}}^m\mho ,{\mathscr {I}}^m\varepsilon )&\le \alpha \frac{\varrho (\mho ,{\mathscr {I}}^m\mho )\varrho (\varepsilon ,{\mathscr {I}}^m\varepsilon )}{\varrho (\mho ,\varepsilon )}\\&\quad + \beta [\varrho (\mho ,{\mathscr {I}}^m\mho )+\varrho (\varepsilon ,{\mathscr {I}}^m\varepsilon )] \\&\quad +~ \gamma \varrho (\mho ,\varepsilon ) + {\mathscr {L}}~ \min \{\varrho (\mho ,{\mathscr {I}}\varepsilon ),\varrho (\varepsilon ,{\mathscr {I}}\mho )\}, \end{aligned} \end{aligned}$$

*for any* $\mho \ne \varepsilon \in {\mathscr {Q}}$* with* $\mho \preceq \varepsilon$,* where* ${\mathscr {L}} \ge 0$,* and* $\alpha , \beta , \gamma \in [0,1)$* with* $0\le \alpha +2\beta +\gamma <1$.* If* $\mho _0 \preceq {\mathscr {I}}^m\mho _0$* for certain* $\mho _0 \in {\mathscr {Q}}$* and* ${\mathscr {I}}^m$* is continuous, then* ${\mathscr {I}}$* has a fixed point*.

### Proof

The proof follows Theorems 1 and 5. $\square$

### Corollary 3

*Let* $({\mathscr {Q}},\varrho ,\preceq )$* be a complete partially ordered metric space. A self map* ${\mathscr {I}}$* has a fixed point, if* $\mho _0 \preceq {\mathscr {I}}^m\mho _0$* for certain* $\mho _0 \in {\mathscr {Q}}$* and satisfies the below contraction condition for some positive integer* *m*,

7 $$\begin{aligned}&\varrho ({\mathscr {I}}^m\mho ,{\mathscr {I}}^m\varepsilon )\le \alpha \frac{\varrho (\mho ,{\mathscr {I}}^m\mho )\varrho (\varepsilon ,{\mathscr {I}}^m\varepsilon )}{\varrho (\mho ,\varepsilon )}\nonumber \\&\quad +\, \beta [\varrho (\mho ,{\mathscr {I}}^m\mho )+\varrho (\varepsilon ,{\mathscr {I}}^m\varepsilon )]+ \gamma \varrho (\mho ,\varepsilon ), \end{aligned}$$

*for all* $\mho \ne \varepsilon \in {\mathscr {Q}}$* with* $\mho \preceq \varepsilon$,* and for some* $\alpha , \beta , \gamma \in [0,1)$* such that* $0\le \alpha +2\beta +\gamma <1$.

### Proof

Setting ${\mathscr {L}}=0$ in Theorem 6, the required proof can be found. $\square$

Let us see the example below.

### Example 6

Let ${\mathscr {Q}}=[0,1]$ with the usual metric and usual order $\le$. Define a map ${\mathscr {I}}:{\mathscr {Q}} \rightarrow {\mathscr {Q}}$ by

$$\begin{aligned} \begin{aligned} {\mathscr {I}}\mho = {\left\{ \begin{array}{ll} 0, \quad &{} if~ \mho \in [0, \frac{1}{6}],\\ \frac{1}{6}, \quad &{} if~ \mho \in (\frac{1}{6},1], \end{array}\right. } \end{aligned} \end{aligned}$$

then ${\mathscr {I}}$ is discontinuous and is not satisfying condition (1) for each $\alpha , \beta , \gamma \in [0,1)$ with $0 \le \alpha +2\beta +\gamma <1$ where as $\mho =\frac{1}{6}, \varepsilon =1$. But ${\mathscr {I}}^2(\mho )=0$ for all $\mho \in [0,1]$ and ${\mathscr {I}}^2$ fulfill all assumptions of Theorem 6. Therefore, ${\mathscr {I}}^2$ has a unique fixed point $0 \in {\mathscr {Q}}$.

### Generalized Rational Contraction Results

#### Theorem 7

*Suppose* $({\mathscr {Q}},\varrho ,\preceq )$* is a complete partially ordered metric space. A non-decreasing continuous self map* ${\mathscr {I}}$* on* ${\mathscr {Q}}$* satisfies*

8 $$\begin{aligned} \begin{aligned} \varrho ({\mathscr {I}}\mho ,{\mathscr {I}}\varepsilon )\le {\left\{ \begin{array}{ll} &{} \lambda \varrho (\mho ,\varepsilon ) +\theta \left[ \varrho (\mho ,{\mathscr {I}}\mho )+\varrho (\varepsilon ,{\mathscr {I}}\varepsilon ) \right] \\ &{}~~+ \mu \frac{\varrho (\mho ,{\mathscr {I}}\mho ) \varrho (\mho ,{\mathscr {I}}\varepsilon )+\varrho (\varepsilon ,{\mathscr {I}}\mho ) \varrho (\varepsilon ,{\mathscr {I}}\varepsilon )}{\varrho (\varepsilon ,{\mathscr {I}}\mho )+\varrho (\mho ,{\mathscr {I}}\varepsilon )} ~~~~, if A \ne 0 \\ &{} 0 ~~~~~~~~~~~~~~~~~~~~~~~~~~~~~~~~~~~~~~~~~~~~, if A = 0 \end{array}\right. } \end{aligned} \end{aligned}$$

*for any* $\mho \ne \varepsilon \in {\mathscr {Q}}$* with* $\varepsilon \preceq \mho$,* where* $A=\varrho (\varepsilon ,{\mathscr {I}}\mho )+\varrho (\mho ,{\mathscr {I}}\varepsilon )$* and,* $\lambda , \theta , \mu$* are non-negative reals such that* $0\le \lambda +2\theta +\mu <1$.* If* $\mho _0 \preceq {\mathscr {I}}\mho _0$* for certain* $\mho _0 \in {\mathscr {Q}}$,* then* ${\mathscr {I}}$* has a fixed point*.

#### Proof

The proof is trivial, if $\mho _0 = {\mathscr {I}}\mho _0$. Suppose not, $\mho _0 \prec {\mathscr {I}}\mho _0$ and then the non-decreasing property of ${\mathscr {I}}$, we acquire that

9 $$\begin{aligned} \mho _0 \prec {\mathscr {I}}\mho _0 \preceq {\mathscr {I}}^2 \mho _0 \preceq ...\preceq {\mathscr {I}}^n \mho _0\preceq {\mathscr {I}}^{n+1}\mho _0 \preceq ...~. \end{aligned}$$

If $\mho _{n_0}=\mho _{n_0+1}$ for certain $n_0 \in {\mathbb {N}}$, then $\mho _{n_0}$ is a fixed point of ${\mathscr {I}}$ from (9). Assume, $\mho _n \ne \mho _{n+1} (n \ge 0)$. From (9), $\mho _n$ and $\mho _{n-1}$ are comparable for each $n \in {\mathbb {N}}$ then we have the discussion below in subsequent cases.

**Case 1:** If $A=\varrho (\mho _{n-1},{\mathscr {I}}\mho _n)+\varrho (\mho _n,{\mathscr {I}}\mho _{n-1}) \ne 0$, then (8) implies that,

$$\begin{aligned} \begin{aligned} \varrho (\mho _{n+1},\mho _n)&=\varrho ({\mathscr {I}}\mho _n,{\mathscr {I}}\mho _{n-1})\\&\le \lambda \varrho (\mho _n,\mho _{n-1}) +\theta \left[ \varrho (\mho _n,{\mathscr {I}}\mho _n)+\varrho (\mho _{n-1},{\mathscr {I}}\mho _{n-1}) \right] \\&\quad +\,\mu \frac{\varrho (\mho _n,{\mathscr {I}}\mho _n) \varrho (\mho _n,{\mathscr {I}}\mho _{n-1})+\varrho (\mho _{n-1},{\mathscr {I}}\mho _n) \varrho (\mho _{n-1},{\mathscr {I}}\mho _{n-1})}{\varrho (\mho _{n-1},{\mathscr {I}}\mho _n)+\varrho (\mho _n,{\mathscr {I}}\mho _{n-1})}, \end{aligned} \end{aligned}$$

which implies that,

$$\begin{aligned} \begin{aligned} \varrho (\mho _{n+1},\mho _n)&\le \lambda \varrho (\mho _n,\mho _{n-1}) +\theta \left[ \varrho (\mho _n,\mho _{n+1})+\varrho (\mho _{n-1},\mho _n) \right] \\&\quad +\,\mu \frac{\varrho (\mho _n,\mho _{n+1}) \varrho (\mho _n,\mho _n)+\varrho (\mho _{n-1},\mho _{n+1}) \varrho (\mho _{n-1},\mho _n)}{\varrho (\mho _{n-1},\mho _{n+1})+\varrho (\mho _n,\mho _n)}. \end{aligned} \end{aligned}$$

Thus,

$$\begin{aligned}&\varrho (\mho _{n+1},\mho _n) \le \lambda \varrho (\mho _n,\mho _{n-1})\\&\quad +\,\theta \left[ \varrho (\mho _n,\mho _{n+1})+\varrho (\mho _{n-1},\mho _n) \right] +\mu \varrho (\mho _{n-1},\mho _n). \end{aligned}$$

Hence,

$$\begin{aligned} \varrho (\mho _{n+1},\mho _n) \le \left( \frac{\lambda +\theta +\mu }{1-\theta }\right) \varrho (\mho _{n-1},\mho _n). \end{aligned}$$

Inductively, we get

$$\begin{aligned} \varrho (\mho _{n+1},\mho _n) \le \hbar ^n \varrho (\mho _1,\mho _0), \end{aligned}$$

here $\hbar = \frac{\lambda +\theta +\mu }{1-\theta }<1$. Also, by the triangular inequality of *d*, for $m \ge n$

$$\begin{aligned} \begin{aligned} \varrho (\mho _m,\mho _n)&\le \varrho (\mho _m,\mho _{m-1})+\varrho (\mho _{m-1},\mho _{m-2})+...+\varrho (\mho _{n+1},\mho _n) \\&\le \frac{\hbar ^n}{1-\hbar }\varrho (\mho _1, \mho _0), \end{aligned} \end{aligned}$$

which implies that, $\varrho (\mho _m, \mho _n) \rightarrow 0$ as $m,n \rightarrow \infty$. Thus, $\{\mho _n\} \subset {\mathscr {Q}}$ is a Chachy sequence and converges to $\zeta \in {\mathscr {Q}}$. Besides, the continuity of ${\mathscr {I}}$ gives that,

$$\begin{aligned} {\mathscr {I}}\zeta&= {\mathscr {I}}\left( \lim \limits _{n \rightarrow \infty }\mho _n \right) \\&= \lim \limits _{n \rightarrow \infty }{\mathscr {I}}\mho _n=\lim \limits _{n \rightarrow \infty }\mho _{n+1}=\zeta . \end{aligned}$$

Therefore, $\zeta \in {\mathscr {Q}}$ is a fixed point of ${\mathscr {I}}$.

**Case 2:** If $A=\varrho (\mho _{n-1},{\mathscr {I}}\mho _n)+\varrho (\mho _n,{\mathscr {I}}\mho _{n-1}) = 0$, then $\varrho (\mho _{n+1},\mho _n)=0$. As a result $\mho _n =\mho _{n+1}$, which is a contradiction. Hence, a fixed point $\zeta \in {\mathscr {Q}}$ for ${\mathscr {I}}$ exists. $\square$

#### Example 7

Let us define a self map ${\mathscr {I}}$ on ${\mathscr {Q}}=[0,1]$ with usual metric and usual order $\le$ as

$$\begin{aligned} {\mathscr {I}}\mho =\frac{5}{16(\mho ^2+\mho +\frac{15}{16})}. \end{aligned}$$

Then ${\mathscr {I}}$ has a fixed point in ${\mathscr {Q}}$.

#### Proof

It is evident that ${\mathscr {I}}$ is continuous and non-decreasing in ${\mathscr {Q}}=[0,1]$ and $\mho _0=0\in {\mathscr {Q}}$ such that $\mho _0=0 \le {\mathscr {I}}\mho _0$. For, $\mho \le \varepsilon$,

$$\begin{aligned} \varrho ({\mathscr {I}}\mho ,{\mathscr {I}}\varepsilon )&= \frac{5}{16}\left| \frac{1}{\mho ^2+\mho +\frac{15}{16}}\right. \\&\left. \quad -\frac{1}{\varepsilon ^2+\varepsilon +\frac{15}{16}}\right| \\&=\frac{5}{16}\left| \frac{(\varepsilon +\mho )(\varepsilon -\mho )+(\varepsilon -\mho )}{(\mho ^2+\mho +\frac{15}{16})(\varepsilon ^2+\varepsilon +\frac{15}{16})}\right| \\&=\left| \frac{5(\mho +\varepsilon +1)}{16(\mho ^2+\mho +\frac{15}{16})(\varepsilon ^2+\varepsilon +\frac{15}{16})}\right| ~|\varepsilon -\mho |\\&\le \frac{16}{45}~|\varepsilon -\mho |, \end{aligned}$$

holds for every $\mho ,\varepsilon \in {\mathscr {Q}}$. For $\lambda = \frac{16}{45}$ and $\theta , \mu \in [0,1)$ such that $0\le \lambda +2\theta +\mu <1$, then $\frac{1}{4} \in {\mathscr {Q}}$ is a fixed point of ${\mathscr {I}}$ as all the conditions of Theorem 7 are satisfied. $\square$

Extracting the continuity criteria on ${\mathscr {I}}$ in Theorem 7, we have the following result.

#### Theorem 8

*If* ${\mathscr {Q}}$* has an ordered complete(OC) property in Theorem* 7,* then a non-decreasing mapping* ${\mathscr {I}}$* has a fixed point in* ${\mathscr {Q}}$.

#### Proof

We only claim that $\zeta ={\mathscr {I}}\zeta$. By an ordered complete metrical property of ${\mathscr {Q}}$, we have $\zeta =\sup \{\mho _n\}$, for $n \in {\mathbb {N}}$ as $\mho _n \rightarrow \zeta \in {\mathscr {Q}}$ is a non-decreasing sequence. The non-decreasing property of a map ${\mathscr {I}}$ implies that ${\mathscr {I}}\mho _n \preceq {\mathscr {I}}\zeta$ or, equivalently, $\mho _{n+1} \preceq {\mathscr {I}}\zeta$, for $n \ge 0$. Since, $\mho _0 \prec \mho _1 \preceq {\mathscr {I}}\zeta$ and $\zeta =\sup \{\mho _n\}$ as a result, we get $\zeta \preceq {\mathscr {I}}\zeta$.

Assume $\zeta \prec {\mathscr {I}}\zeta$. From Theorem 7, there is a non-decreasing sequence ${{\mathscr {I}}^n\zeta } \in {\mathscr {Q}}$ with $\lim \limits _{n \rightarrow \infty } {\mathscr {I}}^n\zeta =\varepsilon \in {\mathscr {Q}}$. Again by an ordered complete(OC) property of ${\mathscr {Q}}$, we obtain that $\varepsilon =sup\{{\mathscr {I}}^n\zeta \}$. Furthermore, $\mho _n=T^n\mho _0 \preceq {\mathscr {I}}^n\zeta$, for $n \ge 1$ as a result, $\mho _n \prec {\mathscr {I}}^n\zeta$, $n \ge 1$, since $\mho _n \preceq \zeta \prec {\mathscr {I}}\zeta \preceq {\mathscr {I}}^n\zeta$, for $n \ge 1$ whereas $\mho _n$ and ${\mathscr {I}}^n\zeta$, for $n \ge 1$ are distinct and comparable.

Now we have the discussion below in the subsequent cases.

**Case 1:** If $\varrho ({\mathscr {I}}^n\zeta , {\mathscr {I}}\mho _n)+\varrho (\mho _n, {\mathscr {I}}^{n+1}\zeta ) \ne 0$, then Eq. (8) becomes,

10 $$\begin{aligned} \begin{aligned} \varrho (\mho _{n+1},{\mathscr {I}}^{n+1}\zeta )&= \varrho ({\mathscr {I}}\mho _n,{\mathscr {I}}({\mathscr {I}}^n\zeta ))\\&\le \lambda \varrho (\mho _n,{\mathscr {I}}^n\zeta ) \\&\quad + \, \theta \left[ \varrho (\mho _n,\mho _{n+1}) + \varrho ({\mathscr {I}}^n\zeta , {\mathscr {I}}^{n+1}\zeta ) \right] \\&\quad +\, \mu \frac{\varrho (\mho _n,\mho _{n+1}) \varrho (\mho _n,{\mathscr {I}}^{n+1}\zeta )+\varrho ({\mathscr {I}}^n\zeta ,\mho _{n+1}) \varrho ({\mathscr {I}}^n\zeta ,{\mathscr {I}}^{n+1}\zeta )}{\varrho ({\mathscr {I}}^n\zeta ,\mho _{n+1})+\varrho (\mho _n,{\mathscr {I}}^{n+1}\zeta )}. \end{aligned} \end{aligned}$$

As $n \rightarrow \infty$ in Eq. (10), we get

$$\begin{aligned} \varrho (\zeta ,\varepsilon ) \le \lambda \varrho (\zeta ,\varepsilon ), \end{aligned}$$

as a result we have, $\varrho (\zeta ,\varepsilon )=0$, since $\lambda <1$. Hence, $\zeta =\varepsilon$. In particular, $\zeta =\varepsilon =sup\{{\mathscr {I}}^n\zeta \}$ in consequence, we get ${\mathscr {I}}\zeta \preceq \zeta$, a contradiction. Therefore, ${\mathscr {I}}\zeta =\zeta$.

**Case 2:** If $\varrho ({\mathscr {I}}^n\zeta , {\mathscr {I}}\mho _n)+\varrho (\mho _n, {\mathscr {I}}^{n+1}\zeta ) = 0$, then $\varrho (\mho _{n+1}, {\mathscr {I}}^{n+1}\zeta ) =0$, which implies that, $\varrho (\zeta ,\varepsilon )=0$ as $n \rightarrow \infty$. By following the similar argument in Case 1, we get ${\mathscr {I}}\zeta =\zeta$. $\square$

Now, found some examples below where there is no assurance of a unique fixed point in Theorems 7 and 8.

#### Example 8

Let ${\mathscr {Q}}=\{(1,0), (0,1)\} \subseteq {\mathbb {R}}^2$ with the Euclidean distance ($\varrho$). Define a partial order (${\mathscr {U}}$) in ${\mathscr {Q}}$ as below:

$$\begin{aligned} {\mathscr {U}}:({\mathscr {m}},{\mathscr {n}})\le ({\mathscr {p}},{\mathscr {q}})~ \text {if and only if} ~ {\mathscr {m}}\le {\mathscr {p}} ~\text {and}~{\mathscr {n}} \le {\mathscr {q}}. \end{aligned}$$

Let ${\mathscr {I}}:{\mathscr {Q}} \rightarrow {\mathscr {Q}}$ by ${\mathscr {I}}(\mho ,\zeta )=(\mho ,\zeta )$. Then ${\mathscr {I}}$ have fixed points in ${\mathscr {Q}}$.

#### Proof

It’s obvious that, $({\mathscr {Q}}, \varrho , \le )$ is a complete partially ordered metric space and also, ${\mathscr {I}}$ is a continuous and non-decreasing mapping satisfyingfor every $\lambda , \theta , \mu \in [0,1)$ with $0 \le \lambda +2\theta +\mu <1$ and, $(1,0)\le {\mathscr {I}}((1,0)),(1,0) \in {\mathscr {Q}}$. Thus, all assumptions of Theorem 7 are met. Hence, (1, 0) and (0, 1) are fixed points of ${\mathscr {I}}$. $\square$

#### Example 9

Let $\{(\mho _n,\zeta _n)\} \subseteq {\mathscr {Q}}$ be a non-decreasing sequence which converges to $(\mho ,\zeta )$ in Example 8. Then $\lim \limits _{n \rightarrow \infty } (\mho _n,\zeta _n)=(\mho ,\zeta )$, where $(\mho ,\zeta )$ is an upper bound as well as supreme of all terms of the sequence. Therefore, all assumptions in Theorem 8 are met and, (1, 0) and (0, 1) are the fixed points of ${\mathscr {I}}$ in ${\mathscr {Q}}$.

If ${\mathscr {Q}}$ satisfies the below condition, then the unique fixed point for ${\mathscr {I}}$ exists in Theorems 7 and 8.

11 $$\begin{aligned} \text {for any} ~\varepsilon ,\zeta \in {\mathscr {Q}},~ \text {there exists}~ \mho \in {\mathscr {Q}}~ \text {which} is \,comparable \,to\, ~\varepsilon ~ \text {and}~ \zeta . \end{aligned}$$

#### Theorem 9

*In addition* ${\mathscr {Q}}$* satisfies the condition* (11)* in Theorems* 7* and* 8,* then one can obtains the unique fixed point of* ${\mathscr {I}}$.

#### Proof

We discuss the proof in the following subsequent cases.

**Case 1:** If $\varepsilon \ne \zeta$ are comparable, then we distinguish the below cases again.

(i). If $\varrho (\zeta ,{\mathscr {I}}\varepsilon )+\varrho (\varepsilon ,{\mathscr {I}}\zeta ) \ne 0$ then Eq. (8) follows that,

$$\begin{aligned} \varrho (\varepsilon ,\zeta )&=\varrho ({\mathscr {I}}\varepsilon ,{\mathscr {I}}\zeta )\\&\le \lambda \varrho (\varepsilon ,\zeta ) +\theta \left[ \varrho (\varepsilon ,{\mathscr {I}}\varepsilon )+\varrho (\zeta ,{\mathscr {I}}\zeta ) \right] +\mu \frac{\varrho (\varepsilon ,{\mathscr {I}}\varepsilon ) \varrho (\varepsilon ,{\mathscr {I}}\zeta )+\varrho (\zeta ,{\mathscr {I}}\varepsilon ) \varrho (\zeta ,{\mathscr {I}}\zeta )}{\varrho (\zeta ,{\mathscr {I}}\varepsilon )+\varrho (\varepsilon ,{\mathscr {I}}\zeta )} \\&\le \lambda d(\varepsilon ,\zeta ) +\theta \left[ \varrho (\varepsilon ,\varepsilon )+\varrho (\zeta ,\zeta ) \right] \\&\quad +\mu \frac{\varrho (\varepsilon ,\varepsilon ) \varrho (\varepsilon ,\zeta )+\varrho (\zeta ,\varepsilon ) \varrho (\zeta ,\zeta )}{\varrho (\zeta ,\varepsilon )+\varrho (\varepsilon ,\zeta )} \\&\le \lambda \varrho (\varepsilon ,\zeta ), \end{aligned}$$

a contradiction as $\lambda <1$. Therefore, $\varepsilon =\zeta$.

(ii). If $\varrho (\zeta ,{\mathscr {I}}\varepsilon )+\varrho (\varepsilon ,{\mathscr {I}}\zeta ) = 0$, then $\varrho (\varepsilon ,\zeta )=0$, a contradiction to $\varepsilon \ne \zeta$. Hence, $\varepsilon =\zeta$.

**Case 2:** Suppose $\varepsilon$, $\zeta$ are not comparable, then from (11) there exists $\mho \in {\mathscr {Q}}$ is comparable to $\varepsilon$, $\zeta$. Besides, the monotone property suggest that ${\mathscr {I}}^n\mho$ is comparable to ${\mathscr {I}}^n\varepsilon =\varepsilon$ and ${\mathscr {I}}^n\zeta =\zeta$ for $n\in {\mathbb {N}}$.

If ${\mathscr {I}}^{n_0}\mho =\varepsilon$, for certain $n_0 \ge 1$, then $\{{\mathscr {I}}^n \mho : n \ge n_0\}$ is a constant sequence, since $\varepsilon$ is a fixed point. As a result, we get $\lim \limits _{n \rightarrow \infty } {\mathscr {I}}^n\mho =\varepsilon$. Assume, if ${\mathscr {I}}^n\mho \ne \varepsilon$ for $n \ge 1$ then the following subcases, we have

(i). If $\varrho ({\mathscr {I}}^{n-1}\varepsilon , {\mathscr {I}}^n\mho )+\varrho ({\mathscr {I}}^{n-1}\mho , {\mathscr {I}}^n\varepsilon ) \ne 0$, then for $n \ge 2$, (8) becomes,

$$\begin{aligned} \begin{aligned} \varrho ({\mathscr {I}}^n \mho ,\varepsilon )&=\varrho ({\mathscr {I}}^n\mho , {\mathscr {I}}^n \varepsilon )\\&\le \lambda \varrho ({\mathscr {I}}^{n-1}\mho ,\varepsilon ) \\&\quad +\theta \left[ \varrho ({\mathscr {I}}^{n-1}\mho ,{\mathscr {I}}^n\mho )+\varrho (\varepsilon ,\varepsilon ) \right] \\&\quad +\mu \frac{\varrho ({\mathscr {I}}^{n-1}\mho ,{\mathscr {I}}^n \mho ) \varrho ({\mathscr {I}}^{n-1} \mho ,\varepsilon )+\varrho (\varepsilon , {\mathscr {I}}^n\mho ) \varrho (\varepsilon ,\varepsilon )}{\varrho ({\mathscr {I}}^n \mho ,\varepsilon )+\varrho (\varepsilon ,{\mathscr {I}}^{n-1}\mho )} \\&\le \lambda \varrho ({\mathscr {I}}^{n-1}\mho ,\varepsilon ) +\theta \left[ \varrho ({\mathscr {I}}^{n-1}\mho ,\varepsilon )+\varrho (\varepsilon ,{\mathscr {I}}^n\mho ) \right] \\&\quad +\mu \varrho ({\mathscr {I}}^{n-1}\mho ,\varepsilon ). \end{aligned} \end{aligned}$$

Thus,

$$\begin{aligned} \varrho ({\mathscr {I}}^n \mho ,\varepsilon ) \le \left( \frac{\lambda +\theta +\mu }{1-\theta } \right) \varrho ({\mathscr {I}}^{n-1}\mho , \varepsilon ). \end{aligned}$$

Inductively, we get

12 $$\begin{aligned} \varrho ({\mathscr {I}}^n \mho ,\varepsilon ) \le \left( \frac{\lambda +\theta +\mu }{1-\theta } \right) ^n \varrho (\mho , \varepsilon ), \end{aligned}$$

which results in ${\mathscr {I}}^n\mho \rightarrow \varepsilon$ as $n \rightarrow \infty$ in Eq. (12). As by the same argument, we obtain that ${\mathscr {I}}^n \mho \rightarrow \zeta$ as $n \rightarrow \infty$. The result of uniqueness implies that, $\varepsilon =\zeta$.

(ii). If $\varrho ({\mathscr {I}}^{n-1}\varepsilon , {\mathscr {I}}^n\mho )+\varrho ({\mathscr {I}}^{n-1}\mho , {\mathscr {I}}^n\varepsilon ) = 0$, then $\varrho ({\mathscr {I}}^n\mho ,\varepsilon )=0$. Therefore, $\lim \limits _{n \rightarrow \infty } {\mathscr {I}}^n \mho =\varepsilon$. Also from the similar argument, we acquire that, $\lim \limits _{n \rightarrow \infty } {\mathscr {I}}^n \mho =\zeta$. Hence, $\varepsilon =\zeta$. $\square$

#### Example 10

Let $C[0,1]=\{\mho :[0,1]\rightarrow {\mathbb {R}}, \text {continuous}\}$ be a space with the partial order

$$\begin{aligned} \mho \le \zeta ~ \text {if and only if}~ \mho (t)\le \zeta (t),~ \text {for}~t\in [0,1], \end{aligned}$$

and let the metric be

$$\begin{aligned} \varrho (\mho ,\zeta )=\sup \{|\mho (t)-\zeta (t)|:t\in [0,1]\}, \end{aligned}$$

satisfies the condition (8) and $\max (\mho ,\zeta )(t)=\max \{\mho (t),\zeta (t)\}$ is continuous. Furthermore, $(C[0,1], \le )$ satisfies the condition (11) and hence the uniqueness.

#### Example 11

Let ${\mathscr {Q}}=\{(0,0),(\frac{1}{2},0),(0,1)\}$ be a subset of ${\mathbb {R}}^2$ with the order $\le$ defined by: for $(\mho _1, \zeta _1),(\mho _2, \zeta _2)\in {\mathscr {Q}}$ with $(\mho _1, \zeta _1)\le (\mho _2, \zeta _2)$ if and only if $\mho _1 \le \mho _2$ and $\zeta _1 \le \zeta _2$. A metric $\varrho :{\mathscr {Q}}\times {\mathscr {Q}} \rightarrow {\mathbb {R}}$ is defined by

$$\begin{aligned} \varrho ((\mho _1, \zeta _1), (\mho _2, \zeta _2))=\max \{|\mho _1-\mho _2|, |\zeta _1-\zeta _2|\}. \end{aligned}$$

A self map ${\mathscr {I}}$ on ${\mathscr {Q}}$ is defined by ${\mathscr {I}}(0,0)=(0,0)$, ${\mathscr {I}}(0,1)=(\frac{1}{2},0)$ and ${\mathscr {I}}(\frac{1}{2},0)=(0,0)$. Therefore, all the assumptions of Theorems 7, 8 and 9 are met and hence, ${\mathscr {I}}$ has a unique fixed point $(0,0) \in {\mathscr {Q}}$.

#### Remark 1

- Theorems 2.1, 2.2 and 2.3 of [20] can be found from Theorems 7, 8 and 9 by setting $\theta =\mu =0$.
- By replacing $\theta =0$ in Theorems 7, 8 and 9, we obtain Theorems 15, 16 and 18 of [26].
- Theorem 20 of [26] can get by putting $\lambda =\theta =0$ in Theorem 7.

Some consequences from Section 0.1 can get by taking $\lambda =0$ and $\theta =0$.

#### Theorem 10

*Suppose* $({\mathscr {Q}},\varrho ,\preceq )$* is a complete partially ordered metric space. A non-decreasing self map* ${\mathscr {I}}$* on* ${\mathscr {Q}}$* satisfies the below contraction condition for every* $\mho \ne \varepsilon \in {\mathscr {Q}}$* with* $\varepsilon \preceq \mho$* then* ${\mathscr {I}}$* has a fixed point, if* $\mho _0 \preceq {\mathscr {I}}\mho _0$,* for certain* $\mho _0 \in {\mathscr {Q}}$.

13 $$\begin{aligned} \begin{aligned} \varrho ({\mathscr {I}}\mho ,{\mathscr {I}}\varepsilon )\le {\left\{ \begin{array}{ll} \theta \left[ \varrho (\mho ,{\mathscr {I}}\mho )+\varrho (\varepsilon ,{\mathscr {I}}\varepsilon ) \right] +\mu \frac{\varrho (\mho ,{\mathscr {I}}\mho ) \varrho (\mho ,{\mathscr {I}}\varepsilon )+\varrho (\varepsilon ,{\mathscr {I}}\mho ) \varrho (\varepsilon ,{\mathscr {I}}\varepsilon )}{\varrho (\varepsilon ,{\mathscr {I}}\mho )+\varrho (\mho ,{\mathscr {I}}\varepsilon )} , \quad &{} if A \ne 0 \\ 0, \quad &{} if A = 0 \end{array}\right. } \end{aligned} \end{aligned}$$

*where* $A=\varrho (\varepsilon ,{\mathscr {I}}\mho )+\varrho (\mho ,{\mathscr {I}}\varepsilon )$,* and* $\theta , \mu$* are non-negative reals with* $0\le 2\theta +\mu <1$* and either* ${\mathscr {I}}$* is continuous or* ${\mathscr {Q}}$* has an ordered complete(OC) property*.

#### Theorem 11

*A non-decreasing self map* ${\mathscr {I}}$* on* ${\mathscr {Q}}$,* where* $({\mathscr {Q}},\varrho ,\preceq )$* be a complete partially ordered metric space has a fixed point, if it satisfies the below contraction condition for every* $\mho \ne \varepsilon \in {\mathscr {Q}}$* with* $\varepsilon \preceq \mho$* and* $\mho _0 \preceq {\mathscr {I}}\mho _0$,* for certain* $\mho _0 \in {\mathscr {Q}}$.

14 $$\begin{aligned} \begin{aligned} \varrho ({\mathscr {I}}\mho ,{\mathscr {I}}\varepsilon )\le {\left\{ \begin{array}{ll} &{} \lambda \varrho (\mho ,\varepsilon )+\mu \frac{\varrho (\mho ,{\mathscr {I}}\mho ) \varrho (\mho ,{\mathscr {I}}\varepsilon )+\varrho (\varepsilon ,{\mathscr {I}}\mho ) \varrho (\varepsilon ,{\mathscr {I}}\varepsilon )}{\varrho (\varepsilon ,{\mathscr {I}}\mho )+\varrho (\mho ,{\mathscr {I}}\varepsilon )} ~~~~~~, if A \ne 0 \\ &{} 0 ~~~~~~~~~~~~~~~~~~~~~~~~~~~~~~~~~~~~~~~~~~~~~~~~~~~~~~, if A = 0 \end{array}\right. } \end{aligned} \end{aligned}$$

*where* $A=\varrho (\varepsilon ,{\mathscr {I}}\mho )+\varrho (\mho ,{\mathscr {I}}\varepsilon )$* and* $\lambda , \mu$* are non-negative reals with* $0\le \lambda +\mu <1$* and either* ${\mathscr {I}}$* is continuous or* ${\mathscr {Q}}$* has an ordered complete(OC) property*.

Besides, a unique fixed point for ${\mathscr {I}}$ can be obtained from Theorems 10 and 11, if ${\mathscr {Q}}$ satisfies the condition (11).

#### Theorem 12

*Suppose* $({\mathscr {Q}},d,\preceq )$* is a complete partially ordered metric space. A nondecreasing self map* ${\mathscr {I}}$* on* ${\mathscr {Q}}$* satisfies the condition* (1)* (or (*5*)) for some* $\mho _0 \in {\mathscr {Q}}$* with* $\mho _0\succeq {\mathscr {I}}\mho _0$,* and is either continuous or* ${\mathscr {Q}}$* satisfies*

$$\begin{aligned} \text {if a non-increasing sequence}~ \{\mho _n\} \rightarrow \mho ~ \text {in}~ {\mathscr {Q}},~ \text {then}~ \mho =\inf \{\mho _n\}. \end{aligned}$$

*Then*${\mathscr {I}}$* has a fixed point in* ${\mathscr {Q}}$.

#### Proof

The procedure of the proof follows Theorems 7 and 8. $\square$

#### Theorem 13

*Condition* (11)* gives the uniqueness of a fixed point of* ${\mathscr {I}}$* in Theorem* 12.

#### Remark 2

In [20], instead of condition (11), the authors use the following weaker condition:

15 $$\begin{aligned}&{\text {if a non-decreasing (non-increasing) sequence}}\, \{\mho _n\} \rightarrow \mho\,  {\text {in}} {\mathscr {Q}},\, {\text {then}} \\ {}&\mho _n \preceq \mho (\mho \preceq \mho _n), {\text {for all}}\, n\in {\mathbb {N}}. \end{aligned}$$

we have not been able to prove Theorem 1 and 8 and its consequences using (15).

We use the following definitions in the upcoming corollaries.

#### Definition 5

Let ${\mathscr {Q}}$ be a nonempty set and $\mho _{0}\in {\mathscr {Q}}$. Let $\mho _{0}$. The orbit of $\mho _{0}$ is defined by ${\mathscr {O}}(\mho _{0})=\{\mho _{0},{\mathscr {I}} \mho _{0},{\mathscr {I}}^2\mho _{0},...\}$.

#### Definition 6

Let $({\mathscr {Q}},\varrho )$ be a metric space and ${\mathscr {I}}:{\mathscr {Q}} \rightarrow {\mathscr {Q}}$. ${\mathscr {Q}}$ is said to be ${\mathscr {I}}$-orbitally complete if every Cauchy sequence in ${\mathscr {O}}(\mho )$, $\mho \in {\mathscr {Q}}$, converges to a point in ${\mathscr {Q}}$.

#### Definition 7

Let $({\mathscr {Q}},\varrho )$ be a metric space and ${\mathscr {I}}:{\mathscr {Q}} \rightarrow {\mathscr {Q}}$. ${\mathscr {I}}$ is said to be orbitally continuous at $\mu \in {\mathscr {Q}}$ if ${\mathscr {I}} \mho _{n} \rightarrow {\mathscr {I}}\mu$ as $n \rightarrow \infty$ whenever $\mho _{n} \rightarrow \mu$ as $n \rightarrow \infty$.

Now a consequence of the main result in terms integral type contractions for an orbitally complete partially ordered metric space is as follows.

#### Corollary 4

*Let* $({\mathscr {Q}},\varrho ,\preceq )$* be a *${\mathscr {I}}$*-orbitally complete partially ordered metric space. A non-decreasing self map* ${\mathscr {I}}$* on* ${\mathscr {Q}}$* satisfies,*

16 $$\begin{aligned} \begin{aligned} \int _{0}^{\varrho ({\mathscr {I}}\mho ,{\mathscr {I}}\varepsilon )}d\Lambda&\le \alpha \int _{0}^{\frac{\varrho (\mho ,{\mathscr {I}}\mho ) \varrho (\varepsilon ,{\mathscr {I}}\varepsilon )}{\varrho (\mho ,\varepsilon )}} d\Lambda \\&\quad + \beta \int _{0}^{\varrho (\mho ,{\mathscr {I}}\mho )+\varrho (\varepsilon ,{\mathscr {I}}\varepsilon )}d\Lambda + \gamma \int _{0}^{\varrho (\mho ,\varepsilon )}d\Lambda \\&\quad +{\mathscr {L}} \int _{0}^{\min \{\varrho (\mho ,{\mathscr {I}}\varepsilon ),\varrho (\varepsilon ,{\mathscr {I}}\mho )\}} d\Lambda , \end{aligned} \end{aligned}$$

*for every* $\mho \ne \varepsilon \in {\mathscr {Q}}$* with* $\mho \preceq \varepsilon$* and there exist* $\alpha , \beta , \gamma \in [0,1)$* with* $0<\alpha +2\beta +\gamma <1$,* and* ${\mathscr {L}} \ge 0$.* If* $\mho _0 \preceq {\mathscr {I}}\mho _0$,* for certain* $\mho _0 \in {\mathscr {Q}}$,* then* ${\mathscr {I}}$* has at least one fixed point in* ${\mathscr {Q}}$.

Similarly, the following result is the consequence of Corollary 1.

#### Corollary 5

*A non-decreasing continuous self-map* ${\mathscr {I}}$* on* ${\mathscr {Q}}$* satisfies the below condition for every* $\mho \ne \varepsilon \in {\mathscr {Q}}$* with* $\mho \preceq \varepsilon$,* then* ${\mathscr {I}}$* has a fixed point, if* $\mho _0 \preceq {\mathscr {I}}\mho _0$* for certain* $\mho _0 \in {\mathscr {Q}}$.

17 $$\begin{aligned} \begin{aligned} \int _{0}^{\varrho ({\mathscr {I}}\mho ,{\mathscr {I}}\varepsilon )}d\Lambda&\le \alpha \int _{0}^{\frac{\varrho (\mho ,{\mathscr {I}}\mho ) \varrho (\varepsilon ,{\mathscr {I}}\varepsilon )}{\varrho (\mho ,\varepsilon )}} d\Lambda \\&\quad + \beta \int _{0}^{\varrho (\mho ,{\mathscr {I}}\mho )+\varrho (\varepsilon ,{\mathscr {I}}\varepsilon )}d\Lambda \\&\quad + \gamma \int _{0}^{d(\mho ,\varepsilon )} d\Lambda , \end{aligned} \end{aligned}$$

*where* $$\alpha , \beta , \gamma \in [0,1)$$* with* $$0<\alpha +2\beta +\gamma <1$$.

## Limitations

In complete partially ordered metric space, the existence of a fixed point of a self mapping satisfying generalized contraction of rational type is discussed. The uniqueness of a fixed point of the mapping is also obtained under an order relation in the space. Suitable examples are given at all possible stages to support the new findings. Some of these results are generalized and extended the well-known results in an ordered metric space. Also a result is widen from general metric space to partially ordered metric spaces with suitable example. A few consequences of the main results in terms of integral contractions are presented at the end.

- The results can be extended for a mapping in partially ordered *b*-metric space to acquire a fixed point.
- We can also obtain a coincidence point, common fixed point, coupled fixed point and coupled common fixed points by involving two mappings of the contraction conditions in partially ordered *b*-metric with required topological properties like monotone non-decreasing, mixed monotone, compatible etc.

## Data Availability

Not applicable.
